# The effect of prenatal education on fear of childbirth, pain intensity during labour and childbirth experience: a scoping review using systematic approach and meta-analysis

**DOI:** 10.1186/s12884-023-05867-0

**Published:** 2023-07-27

**Authors:** Zohreh Alizadeh-Dibazari, Somayeh Abdolalipour, Mojgan Mirghafourvand

**Affiliations:** 1 Midwifery Department, Faculty of Nursing and Midwifery, Students’ Research Committee, Tabriz University of Medical Sciences, Tabriz, Iran; 2 Social Determinants of Health Research Center, Faculty of Nursing and Midwifery, Tabriz University of Medical Sciences, Tabriz, Iran

**Keywords:** Education, Labour, Childbirth, Fear of childbirth, Pain, Childbirth experience, Depression, Anxiety, Maternal attachment, Maternal bonding

## Abstract

**Background:**

Antenatal education provides parents with strategies for pregnancy, childbirth, and parenthood. There is not enough evidence of the positive effect of prenatal education on childbirth and maternal outcomes. The present scoping review using a systematic approach, evaluates the effectiveness of prenatal education on fear of childbirth, pain intensity during labour, childbirth experience, and postpartum psychological health.

**Methods:**

We used Google Scholar and systematically reviewed databases such as PubMed, Web of Science, Cochrane, Scopus, and SID (Scientific Information Database). Randomized controlled and quasi-experimental trials examining the effect of structured antenatal education and routine prenatal care compared to routine prenatal care were reviewed. The participants included pregnant women preferring a normal vaginal delivery and had no history of maternal or foetal problems. The outcomes considered in this study included fear of childbirth, pain intensity during labour, childbirth experience (as primary outcomes) and postpartum psychological health (as secondary outcomes). The grading of Recommendations Assessment, Development and Evaluation (GRADE) approach was used to evaluate the quality of evidence.

**Results:**

Overall, 3242 studies were examined, of which 18 were qualified for the final analysis. The meta-analysis showed that providing prenatal education and routine care compared to only routine care may decrease the fear of childbirth, postpartum depression, and pain intensity during labour. However, we found no study examining the outcome of the childbirth experience. In addition, the inconsistency of included studies prevented conducting a meta-analysis on the rest of the outcomes.

**Conclusions:**

Our investigations showed that there are very few or no studies on the effect of prenatal education on outcomes such as childbirth experience, postpartum anxiety, and maternal attachment, and the existing studies on the effect of prenatal education on outcomes such as the fear of childbirth, postpartum depression, and pain intensity during labour lack sufficient quality to make definitive conclusions. Therefore, high-quality, randomized trials with a more extensive sample size are suggested to provide clear reports to make definitive decisions.

**Prospero ID:**

CRD42022376895.

**Supplementary Information:**

The online version contains supplementary material available at 10.1186/s12884-023-05867-0.

## Introduction

Pregnancy and childbirth are among women’s most important experiences. Pregnancy leads to significant challenges, such as physical changes, psychological stress, and changing roles. In addition, childbirth has physical, psychological, emotional, social, and cultural dimensions [1]. Much evidence confirms the relationship between pregnancy and psychological problems such as fear, anxiety, and depression [2]. Fear of childbirth commonly leads to prolonged labour [3], more severe pain experience [4], a negative impact on the birth experience [5], and increasing requests for elective caesarean delivery [6, 7]. Anxiety and self-efficacy for childbirth are inversely related. The pregnant woman’s low self-efficacy increases their perception of childbirth pain and, in turn, increases their level of anxiety [8, 9].

There is a relationship between anxiety levels experienced during pregnancy and poor mother-infant bonding quality during pregnancy and after birth [10]. The World Health Organization (WHO) has requested preparation for birth as an essential component of prenatal care [11].

Prenatal education worldwide is essential in preparing couples for pregnancy, childbirth, and parenthood [12]. Prenatal education generally offers expectant parents strategies for coping with pregnancy, childbirth, and parenthood [13]. Its specific goals include increasing knowledge, growing mothers’ confidence in giving birth and parenthood, positively affecting childbirth experiences, promoting breastfeeding, and improving newborn care, postpartum care, and parenting skills in the postpartum period [14, 15].

Prenatal education classes in many countries are based on basic philosophies such as Dick-Read, Bradley, Lamaze, and Hypnosis. Pregnancy education classes discuss many topics, including health during pregnancy, physiological pregnancy changes, complaints and high-risk situations, nutrition during pregnancy, the mother’s role and relationship, childbirth preparation, pain control during childbirth, postpartum period and breastfeeding, newborn care, and pregnancy exercise [16]. American College of Obstetricians and Gynecologists (ACOG) suggests that exercise during pregnancy improves women’s health and prevents gestational diabetes and excessive weight gain [17].

Studies on the effect of prenatal education on childbirth and parenthood show that this education reduces the fear of childbirth [12, 18–20], anxiety at birth [17, 20, 21], depression [20], the perceived childbirth pain [22] and increases childbirth self-efficacy [12, 18, 19, 21, 22]. Although some studies do not confirm the effect of prenatal education on the childbirth experience [14] and parental attachment [12], Toosi et al. (2014) found that relaxation training during pregnancy increases maternal attachment [23], and Abbasi et al. (2013) found that maternal attachment promotion training was successful [24]. Gagnon and Sandall (2007) performed a meta-analysis on 2284 women participating in childbirth preparation courses. They concluded that the effect of prenatal education on women’s level of awareness and anxiety, sense of self-control, perceived labour pain, and social and emotional adjustment was not significant [15].

Due to limited healthcare resources, policymakers should make informed and scientific decisions about healthcare priorities [25]. A review is needed to evaluate the effectiveness of prenatal education compared to providing no education. Therefore, the present scoping review using a systematic approach evaluates the effects of antenatal education on fear of childbirth, pain intensity during labour, childbirth experience, and postpartum psychological health.

## Methods

We used Cochrane Handbook for Systematic Reviews of Interventions [26] and the PRISMA statement and registered the study on the International Prospective Register of Systematic Reviews (PROSPERO) (Registration number: CRD42022376895).

### Search strategy

Databases such as PubMed, Web of Science, Cochrane, Scopus, SID (Scientific Information Database) and search engine Google Scholar were explored. Search terms were adapted according to each database. The complete search strategy for each database is provided in Appendix 1, and only the clinical trials in English and Persian were searched. This study reviews the article cited in previous research and previous systematic reviews. No restrictions were applied to the publication date. The search was conducted two times: at the beginning of the study and just before the end of the study. No differences were found between the two-time points regarding the included studies.

### Eligibility criteria

This scoping review using a systematic approach examined randomized controlled and quasi-experimental trials in which the intervention group received structured prenatal education and routine prenatal care, and the control group received only routine prenatal care. Structured prenatal education include training classes with various contents such as basic knowledge of pregnancy, care during pregnancy, the process of childbirth, care during the postpartum period and care of newborns, which were presented by trained midwives, nurses, or obstetricians and were held at least four sessions one hourly between 20 and 37 weeks of pregnancy [27]. Routine prenatal care included taking a complete history, performing physical and ultrasound examinations, and giving education. It last about 15–20 min, which is not sufficient time for antenatal education [28].

The participants were pregnant women desiring a normal vaginal delivery (a vaginal delivery, whether or not assisted or induced, usually used to contrast with the delivery by caesarean Sect. [29]) and having no maternal or foetal problems history. This study examined these outcomes: fear of childbirth, pain intensity in the first and second labour phases, and childbirth experience (as primary outcomes) and maternal attachment, postpartum depression, and postpartum anxiety (as secondary outcomes). Valid and reliable tools assessed all outcomes in all included studies. For example, a W-DEQ or other researcher-made questionnaires assessed the fear of childbirth. A numerical rating scale or visual analogue scale assessed pain intensity in the first and second labour phases. The Edinburgh Postnatal Depression Scale (EPDS) was used to assess postpartum depression, and Depression Anxiety and Stress Scale (DASS-21) was used to assess postpartum depression and anxiety.

### Selection of studies

The reviewed articles were selected in two stages. First, two authors (Z.A-D & S.A) independently examined all the titles and abstracts to determine the studies’ eligibility in the systematic search. Second, the full text of the articles obtained in the first stage was evaluated based on the inclusion criteria. Possible disagreements were resolved through discussion or with the help of the third author (M.M).

### Data extraction

A form based on the Cochrane manual for systematic review was designed to contain all the articles’ information [26]. Two researchers (Z.A-D & S.A) independently collected each article’s data, including authors’ names, year of publication, study place, intervention type, comparison group, final sample size, measurement tool, outcomes, and results.

### Quality assessment

When assessing the risk of bias, the selected randomized controlled trials were investigated using the risk of bias-1 approach (ROB-1) [30] regarding random sequence generation, allocation concealment, blinding of participants and personnel, blinding of outcome assessors, selective reporting, and incomplete outcome data. In addition, the selected semi-experimental trials were investigated using the ROBINS-1 approach [31].

Two authors (Z.A-D & S.A) used the Grading of Recommendations Assessment, Development and Evaluation (GRADE) approach to independently evaluate the quality or certainty of evidence in five dimensions: the risk of bias, inconsistency, indirectness, imprecision, and publication bias. Possible disagreements were resolved through discussion with the third author (M.M). All the trials were described and compared regarding demographic information and intervention type when investigating inconsistency. Statistical heterogeneity was checked using the I^2^ statistic and 95% confidence interval (with I^2^ ≥ 60% showing reduced certainty of the evidence due to inconsistency) [32]. In evaluating Indirectness, the study population, type of intervention, control group, and outcomes of included studies were examined to answer the question of the current review [33]. The sufficiency of the participants’ number in included trials for calculating the effect estimate and the confidence interval size around the effect estimate was examined when investigating imprecision [34]. Regarding publication bias, the study size of included trials was examined. Then, the outcomes with more than ten studies were plotted using a funnel plot, with asymmetric funnel plots indicating possible bias [35]. When calculating the evidence quality of the examined outcomes, potential severe concerns about any dimension were resolved by a one-degree reduction of the evidence quality. A two-degree decrease in the quality of evidence was used to determine very severe concerns.

### Synthesis of results

#### Measures of treatment effect

Mean and standard deviation changes of baseline and post-intervention in both groups (intervention group received prenatal education and routine care and control group received only routine care) were calculated. Accordingly, the intervention effects on the continuous outcomes studied in the trials were calculated. Then, a mean difference (95% confidence interval) was used to report the mean change difference. In addition, a standardized mean difference (SMDs) (95% confidence interval) was used to report outcomes using different scales to examine continuous outcomes [36].

#### Data synthesis

Meta-analysis using Review Manager 5.3 was performed to compare the studied outcomes between the intervention and control groups in cases with at least two trials. Previous studies have used different tools to evaluate the outcomes. This study used the random effect method instead of the fixed effect method to calculate the intervention’s effect size on the desired outcome due to high heterogeneity.

## Results

### Results of the search

**Fig. 1:**
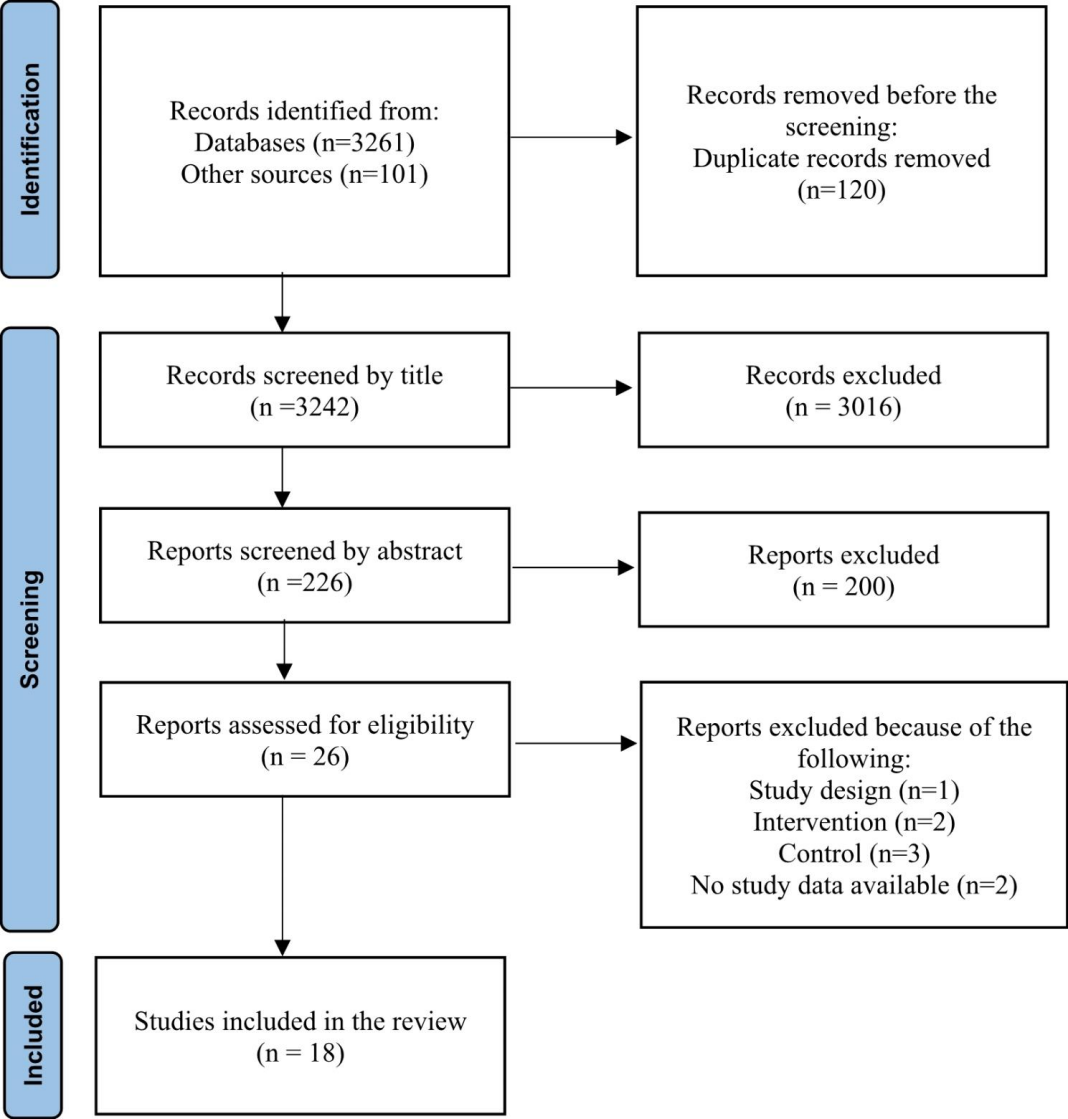
Study flow diagram

The results of the study search strategy are summarized in the PRISMA diagram (Fig. 1). Overall, 3242 studies were examined, of which 3216 did not meet the inclusion criteria and were excluded. Finally, 26 studies were reviewed, of which 18 trials [12, 22, 27, 28, 37–50] were included in the final analysis, which one of them was PhD thesis [46], and the remaining eight trials [51–58] were excluded due to the lack of inclusion criteria.

### Characteristics of included studies

The characteristics of the trials included in the review are summarized in the characteristics table. This table contains information about the study design, the studied groups, intervention types, the blinding type, the follow-up period, the number of participants in each group, the inclusion and exclusion criteria, primary and secondary outcomes, and results (Table 1).

**Table 1:** Characteristics of included studies

Author (s) Location/(year)	Study design	Study groups	Type of intervention/s	Type of blinding	Follow-up period	Number of participants in each group	Health status of participants	Main outcome/s	Secondary outcome/s	Results
Beydokhti et al. Iran/(2020)	RCT^a^	Group 1: Intervention group Group 2: Control group	Intervention group: Educational intervention programme included training sessions for 12 people once a week for 60 to 90 min for four consecutive weeks Control group: Routine pregnancy care	Open-label design	Fourth–sixth week postpartum	Intervention group: 60 Control group: 70	Women in 30–35 weeks of pregnancy, no history of depression, reading and writing literacy, a healthy fetus in ultrasonography, living in the city	Postpartum depression	-	Prevalence of postpartum depression after the intervention was 6.7% and 34% in the intervention and control groups, respectively, and a significant difference was observed between the two groups in terms of depression prevalence (*p* = 0.001)
Calpbinici & Özçirpan. Turkey/(2022)	RCT^a^	Group 1: Intervention group Group 2: Control group	Intervention group: Training Program through Motivational Interview Method in 4 sessions of 45 to 90 min once a week Control group: Routine hospital practices	Open-label design	Within 24 h after delivery	Intervention group:37 Control group: 36	Primiparous pregnant women who were between the 24th to 28th weeks of gestation had no contraindications for vaginal delivery and did not have risks	Fear of childbirth, childbirth self-efficacy, delivery mode	-	It was found that the mean W-DEQ score of the women in the intervention group during the 37th to 40th weeks of gestation was statistically significantly lower than those in the control group
Çankaya & Şimşek. Turkey/(2019)	RCT^a^	Group 1: Intervention group Group 2: Control group	Intervention group: Antenatal education: The sessions were held using simulator models, animation videos, role-playing, creative drama, and slide presentations in classes of 8 to 10 people twice a week for two weeks Control group: Prenatal care services	single-blind	Six^th^ – eight^h^ weeks of postpartum	Antenatal education: 55 Prenatal care services: 57	Nulliparous women older than 18 years of age with a healthy singleton pregnancy in 20 and 32 weeks of gestation with childbirth at full term, having a healthy newborn (born at 38–42 weeks), and not participating in another prenatal Program without mental disorder, high-risk pregnancies, stillbirth, or postpartum complications.	Childbirth self-efficacy; depression, anxiety, and stress symptoms during pregnancy and postpartum; fear of birth during pregnancy and postpartum; and mode of delivery	-	Childbirth self-efficacy (*p* < 0.001), outcome expectancy (*p* < 0.001), and efficacy expectancy (*p* = 0.002) scores the pregnant women receiving antenatal education were significantly higher than those of the control group. Childbirth fear of the women receiving antenatal education was significantly less than the control group (*p* < 0.001).
Dai et al. china/(2021)	RCT^a^	Group 1: Intervention group Group 2: Control group	Intervention group: Simulation-based childbirth education (SBCE) in four 70-minute childbirth education sessions with four to six participants at each session. Control group: Routine prenatal care and health education	Open-label design	After birth	Intervention group: 26 Control group: 30	Primiparous women with a singleton pregnancy, aged 20–35 years, gestational age of 24–32 weeks, having FOC	Fear of childbirth.	Childbirth self-efficacy and birth outcomes, including delivery method, labour duration and Apgar scores	After the SBCE, the mean post-test WDEQ-A score of the intervention group was 60.96 (SD = 14.10), while the score of the control group was 70.79 (SD = 14.85). There was a significant change in the intervention group, up to 14.54 (SD = 11.67, P < 0.001). As for the control group, there was no statistical decrease in FOC scores (mean=-3.23, SD = 12.04, P = 0.152)
Kurdy et al. Egypt/ (2017)	RCT^a^	Group 1: Intervention group Group 2: Control group	Intervention group: antenatal education classes in three 90-minute weekly sessions with ten women in each group by using posters, slide powerpoint presentations, animation videos, and demonstration Control group: Routine antenatal care	Open-label design	Childbirth process (the first and second stages of childbirth)	Intervention group: 52 Control group: 52	Primigravida who: Aged from 20 to 35 years old, was at their 32nd to 34th weeks of gestation, had a singleton vaginal delivery, had no medical or psychological problems	childbirth self-efficacy	Labour pain	The mean score of labour pain during the 1st stage among women in the antenatal education and control groups were 5.08 ± 0.68 & 7.40 ± 0.5, respectively. Also, the mean score of labour pain in the 2nd stage among women in the antenatal education and control groups were 6.52 ± 0.5 & 8.56 ± 0.7, respectively. The two groups had highly significant differences regarding the mean score of labour pain at the 1st and 2nd stages of labour (p = < 0.001).
Firouzbakht et al. Iran/ (2015)	Semi-experimental study	Group 1: Case group Group 2: Control group	Case group: Prenatal education” on the process of childbirth in eight 90-minute weekly sessions Control group: Routine care	Open-label design	Childbirth process	Case group: 63 Control group: 132	Women completed the 5th grade of elementary school, current gestational age of 16–20 weeks, 17–35-year-old, and no contraindication for a vaginal delivery without any complication during pregnancy.	Hospital depression and anxiety, the intensity of pain and intervention in labour		The anxiety level in the case group (who received education) was 14.47 (4.69), and in the control group, it was 16 (4.86) (*P* < 0.001). The pain intensity in the case group was 85.68 (1.85), and in the control group was 90.99 (14.72) (*P* = 0.03).
Gurkan & Ekşi. Turkey/(2017)	Nonrandomized, post-test-control group design	Group 1: Education group Group 2: Control group	Education group: Antenatal education in a 4-week program (12 h) Control group: Routine antenatal care	Open-label design	Sixth month postpartum	Education group = 31 Control group = 34	Women who were aged over 18 years were in the 20th-36th gestational weeks, were primipara, were married, did not have pregnancy- related complications or were not diagnosed with depression in the pre-pregnancy period	Functional status after childbirth and postpartum depression		The mean EPDS score at the 6th postpartum week was 8.3 ± 5.8 in the education group and 8.7 ± 6.2 in the control group. The mean EPDS score in the 6th postpartum month was 5.7 ± 4.7 in the education group and 6.1 ± 5.6 in the control group. EPDS scores were similar within the groups at the 6th week and the 6th month, and there was no significant difference between groups (p > 0.05)
Isbir et al. Turkey/(2017)	Quasi-experimental study.	Group 1: Intervention group Group 2: Control group	Intervention group: Antenatal education in classes with 5–8 women and four 240-minute weekly sessions Control group: Routine prenatal care	Open-label design	Six^th^ –eight^h^ weeks of postpartum	Intervention group: 44 Control group: 46	Nulliparous women, between 20–32 weeks gestation, have no history of pregnancy complications, graduated from at least primary school, and can communicate in Turkish.	Fear of childbirth, maternal self -efficacy and Post-traumatic Stress Disorder Symptoms following childbirth		The difference in fear of birth in the postpartum period between the groups was significant (*p* < 0.05). Women in the the intervention group had significantly lower PTSD symptoms following childbirth than women in the control group in the postpartum period (*p* < 0.01)
Karabulut et al. Turkey/(2016)	a quasi-experimental and prospective study	Group 1: Education group Group 2: Control group	Education group: group education for five weeks once a day for 180 min using relevant appropriate models and figurines, visual instruments and videos, motion, demonstration and interactive education methods Control group: Routine prenatal care	Open-label design	30th − 34th weeks of gestation	Education group: 69 Control group:123	Primipara women aged 18 years and older who could speak and understand Turkish, had a single foetus and were within weeks 24–28 of pregnancy	Women’s adaptation to pregnancy and motherhood and fear of childbirth		There was a significant difference in the levels of AP^b^ between the EG’s post-education measurement and the CG’s second measurement (*P* < 0.001) the EG’s post-education and CG’s second measurement levels of IMR^c^ showed no significant differences (*P* > 0.05). The EG’s post-education and CG’s second measurement levels of FOC^d^ also showed significant differences (*P* < 0.005)
Masoumi et al. Iran/(2016)	RCT^a^	Group 1: Intervention group Group 2: Control group	Intervention group: training preparation for childbirth in 8 sessions of 2 h weekly Control group: routine prenatal education	Open-label design	Childbirth process	Intervention group: 75 Control group:75	Pregnant women with a single fetus, no chronic disease such as diabetes, heart and lung chronic diseases, no infertility, no high-risk pregnancy and no history of psychiatrist visits, do not use specific drugs, gestational age of 20 weeks	Fear of normal vaginal delivery and type of delivery		After the intervention, the mean fear score in the intervention group compared to the control group was significantly reduced (51.7 ± 22.4 vs. 58.7 ± 21.7) (p = 0.007). And mean score in the intervention group after training was lower than before.
Mehrabadi et al. Iran/(2020)	RCT^a^	Group 1: Intervention group Group 2: Control group	Intervention group: Antenatal education in 8 sessions of 90 min Control group: Routine prenatal care	Open-label design	14 day after childbirth	Intervention group: 56 Control group:44	Primipara women with singleton low-risk pregnancies between 20 to 24 gestation weeks with no history of mental illness and with the ability to read and write without previous history of drug and substance abuse	Fear of childbirth,		The comparison of the two intervention and control groups showed that the mean score of the Wijma questionnaire after the intervention was higher in the intervention group compared to the control group (p < 0.001).
Ondieki. Kenya/(2022)	RCT^a^	Group 1: Intervention group Group 2: Control group	Intervention group: Integrated antenatal education module in 5 sessions of 120 min with 7 participants Control group: Routine antenatal care clinic visits	Single blind	37th − 40th weeks of gestation	Intervention group:56 Control group: 53	Primipara women aged 18 to 45 years with singleton low-risk pregnancy between 22 to 26 gestation weeks with no history of mental illness and with the ability to read and write either in English or Kiswahili language without previous history of drug and substance abuse	Fear of childbirth		There was a significant difference in the W-DEQ-A mean scores before (M = 79.89, SD = 10.66) and after the integrated antenatal education (M = 66.75, SD = 19.69, p < 0.001).
Ozcoban et al. Turkey/(2022)	RCT^a^	Group 1: Intervention group 1 Group 2: Intervention group 2 Group 3: Control group	Intervention group 1: Antenatal education focused on improving health literacy in 15 h of education for five weeks with 10–15 participants Intervention group 2: Antenatal education in 15 h of education for five weeks with 10–15 participants Control group: No intervention	Single blind	Five weeks post-education	Intervention group 1: 53 Intervention group 2: 56 Control group: 73	Primiparous pregnant women who volunteered, were 18 years old or over, had no diseases, and were in their second trimesters	Personal Information	Prenatal Self-evaluation, Fear of Childbirth, Postpartum Period Scale, General Self-Efficacy, Turkey Health Literacy Scale	Post-education fear of childbirth scores was significantly higher in the control group compared to the other two groups (*p* = 0.036),
Sanaati et al. Iran/(2018)	RCT^a^	Group 1: Intervention group 1 Group 2: Intervention group 2 Group 3: Control group	Intervention group 1: lifestyle-based education for both the women and their husbands Intervention group 2: lifestyle-based education for only the women in four 60- to 90-min sessions with an_7-day interval (weeks 24–28) with 5–15 participant Control group: Routine care.	Single blind	Six weeks postpartum	Intervention group 1: 62 Intervention group 2: 62 Control group: 63	Pregnant mothers with a gestational age of 24–28 weeks, an uncomplicated singleton pregnancy, an Edinburgh Postnatal Depression Inventory score of lower than 12, first or second pregnancy, at least a secondary education	Postnatal depression and anxiety		When compared with the control group, significant reductions in PPD (adjusted difference: -5.5), state anxiety (-13.6) and trait anxiety (-12.6) scores were observed in the lifestyle education–dyad group, and also significant reductions in PPD (-3.2), state anxiety (-5.8) and trait anxiety (-4.9) scores were observed in the lifestyle education– women only group
Serçekuş & Başkale. Turkey/(2015)	quasi-experimental design	Group 1: Experimental group Group 2: Control group	Experimental group: Antenatal education was provided to groups of four to six couples once a week (120 min) for eight weeks Control group: Routine antenatal care	Open-label design	Six months postpartum	Experimental group: 28 women and their husband Control group: 27 women and their husbands	Women with a gestation of 26–28 weeks, minimum education level of primary school graduation, nulliparous, not at high risk in pregnancy, not attended any other antenatal programme in the antenatal period, give birth at full term, have a healthy newborn and have experienced no postnatal complications	Fear of childbirth, self- efficacy and parental attachment,		The mean W-DEQ score of the women in the experimental group was lower than that of the women in the control group, which indicates that their fear of childbirth was less than that of the women in the control group (p < 0.01). Mean MAI^e^ and PPAQ^f^ scores of the couples in the experimental group and those in the control group showed no significant difference was found between the groups (p > 0.05).
Taheri et al. Iran/(2014)	quasi‑experimental study	Group 1: Intervention group Group 2: Control group	Intervention group: Antenatal education in three60-90 min sessions during a week (including 8–10 persons in each session) Control group: Routine prenatal care	Open-label design	One month after training	Intervention group: 63 Control group: 65	Pregnant women at 24–32 weeks of pregnancy, without medical indications for cesarean sections and the cases of premature delivery or emergency caesarean delivery	Fear of childbirth and self-efficacy		There was a significant difference between two groups in terms of childbirth fear, childbirth expectation and childbirth self-efficacy after (*P* < 0.001) but not before intervention (*P* > 0.05)
Toosi et al. Iran/(2012)	RCT^a^	Group 1: Intervention group Group 2: Control group	Intervention group: Antenatal education in four 90 min sessions weekly Control group: Routine prenatal care	Open-label design.	After intervention	Intervention group: 42 Control group: 42	Primipara women aged 18 to 35 years with singleton low-risk pregnancy between 32 to 35 gestation weeks, minimum education level of primary school graduation, not at high risk in pregnancy with no history of mental illness	Mother-infant attachment		There was no statistically significant difference in the average attachment score in the two groups at the beginning of the study (p = 0.44), but after the study, this difference was significant (p < 0.001).
Uludag et al. Turkey/(2022)	RCT^a^	Group 1: Intervention group Group 2: Control group	Intervention group: Online antenatal childbirth preparation education in groups of 7–8 participants lasted four h weekly for two weeks. Control group: Routine prenatal care	Single-blind		Intervention group: 23 Control group: 21	Women at the age of 18 years or more, the gestation of 24–34 weeks, graduation at least from primary school, nulliparity, not being at high risk in pregnancy, ability to use the application of Microsoft Teams, not having a psychiatric disease and not having attended any other antenatal programmes in the antenatal period.	Worries about labour, fear of birth, Prenatal self-evaluation, fear of COVID-19		The pregnant women in the intervention group were significantly less worried about labour (*p* < 0.05), significantly less afraid of birth ( *p* < 0.05) and significantly less afraid of COVID-19 (*p* < 0.05), significantly more prepared for labour (*p* < 0.01) and had significantly more positive feelings about their wellbeing and their babies’ wellbeing (*p* < 0.05)

^a^ Randomized Controlled Trial; ^b^Acceptance of pregnancy; ^c^Identification with the motherhood role; ^d^Fear of childbirth; ^e^ Maternal attachment inventory; ^f^Postnatal paternal–infant attachment questionnaire

### Included studies

This study systematically reviewed 12 randomized controlled trials [22, 28, 37–40, 44–48, 50] and six semi-experimental trials [12, 27, 41–43, 49] on 2056 pregnant women in Iran, Turkey, China, Egypt, and Kenya. The studies, including 17 articles and one PhD thesis, were published between 2014 and 2022. Two papers were in Persian, and the rest were in English. In all the trials, the control group only received routine prenatal care (taking a complete history, performing physical and ultrasound examinations, and giving education. It lasted about 15–20 min, which was not sufficient time for antenatal education), but the intervention group also received prenatal education. Pregnant women in the intervention group received structured training during pregnancy from trained midwives, nurses, or obstetricians. Different studies had different training classes: the training classes were held between 16 and 36 weeks of pregnancy, each class included 5 to 15 participants, each course included 3 to 8 sessions, each training session lasted 45 to 240 min, and the classes were held one to two times a week. The education included various contents such as physical, functional, and psychological changes during pregnancy, familiarity with the female reproductive system, warning signs during pregnancy, advantages and disadvantages of vaginal childbirth and caesarean section, nutrition and exercise during pregnancy, and stages of labour. Other contents included signs of false labour versus real labour, proper behaviours at the beginning of labour, labour breathing and pain-reducing techniques, natural, physical, and psychological changes after childbirth, after-labour dangerous signs, newborn care, and breastfeeding techniques. The training also included counselling through questions and answers, free discussion on intended topics, mental and muscle exercises, education on appropriate positions for labour and delivery, and appropriate breathing during labour and delivery.

Some specific studies were focused on more educational content. For example, the educational content of studies examining postpartum depression and anxiety was focused more on the self-image and emotional, mental, and psychological health of mothers in the postpartum period [27, 37, 39, 48]. Similarly, studies examining maternal attachment were focused on training mechanisms of mother-and-foetus communication or parents-foetus adaptation methods [12, 50]. Educational classes were held in person in health centres or hospitals, and the contents were presented through lectures, posters, PowerPoint slides, animation videos, and role plays. Uludag et al. in Turkey was the only study being held online due to the Covid-19 epidemic [28]. In addition to in-person training classes, a study examining postpartum depression and anxiety also provided the participants with an educational booklet for study at home, separate ten-minute telephone consultations for mother and father after the delivery, and answering the participants’ questions by phone [48]. The control group in the studied trials received routine care during pregnancy, including taking a complete history, physical examination, and sonography, with the trials lasting 10 to 20 min.

The participants were pregnant women aged 18 to 35 years, with a singleton pregnancy, a gestational age of 14 to 36 weeks, and a desire to have a normal vaginal delivery which in 13 reviewed studies were nulliparous [12, 22, 27, 28, 38–40, 42, 43, 45–47, 50] and in the rest of reviewed studies were multiparous [37, 41, 44, 48, 49]. The participants had consented to participate in the trials. Being nulliparous was not an inclusion criterion in some studies [37, 41, 44, 49], and women with one or two pregnancy histories were also included [48]. Some exclusion criteria in these trials were being unmarried, having a history of psychiatric diseases, drug addiction, and other medical diseases such as gestational hypertension, gestational diabetes, preeclampsia/eclampsia, placenta previa, preterm delivery, and diagnosis of congenital foetal abnormalities or foetal disease. The pregnant mothers and their partners participated in training classes in two studies. In the first one, the mothers and their partners were compared to those in the control group [12]. In the second one, the partners without mothers participated in a training session of 15–25 partners. It compared three groups (e.g., mothers with their trained partners, mothers with their untrained partners, and the control group mothers) regarding the intended outcome [48]. We found no study examining the effect of prenatal education on mothers’ childbirth experience. Two studies examined the effect of prenatal education classes on mothers’ attachment behaviour; one studied the effect of maternal-foetal attachment during pregnancy [50], and the other investigated parental-infant attachment and maternal bonding after delivery [12].

### Excluded studies

The reasons for excluding seven studies from the review were as follows: three studies had provided the same prenatal education to the intervention and the control groups [51, 52, 58], one study was designed as a single-group study before and after the intervention [54], one paper’s full text was out of our reach [53], two paper had used interventions different from those intended in the present study [55, 56], and one paper was in the Indonesian language [57] (Table 2).

**Table 2:** Characteristics of excluded trials and main reasons for exclusion

**Same content of antenatal education in experimental and control groups**	
Badrin 2021	Participants in the control group received antenatal education class sessions.
Bergstrom 2009	Participants in the control group received standard antenatal education focusing on childbirth and parenthood without psychoprophylactic training.
Maimburg 2010	Participants in the control group received different antenatal training programmes, which provided approximately 3 h of lessons on the birth process.
**Differences in study design**	
Koh 2021	Single-group pretest-posttest study design
**No study Data**	
Hay 2022	Lack of access to the full text of the article
**Experimental conditions outside the scope of this review**	
Kuo 2022	Mindfulness practice with childbirth education
Leventhal 1989	Instructions to monitor labour contractions given to parturients
**The language of the article**	
Lumbanraja 2016	Writing the article in Indonesian

### Risk of bias in included studies

**Fig. 2:**
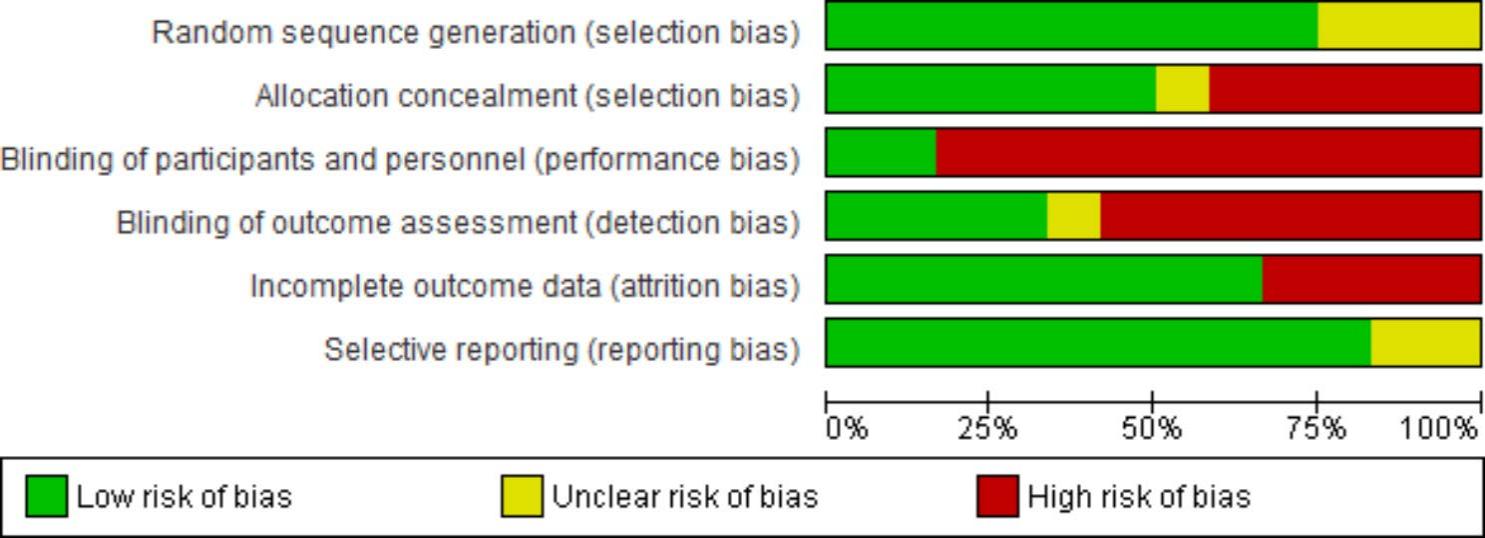
Risk of bias graph. Review authors’ judgments about each risk of bias item presented as percentages across all included studies

**Fig. 3:**
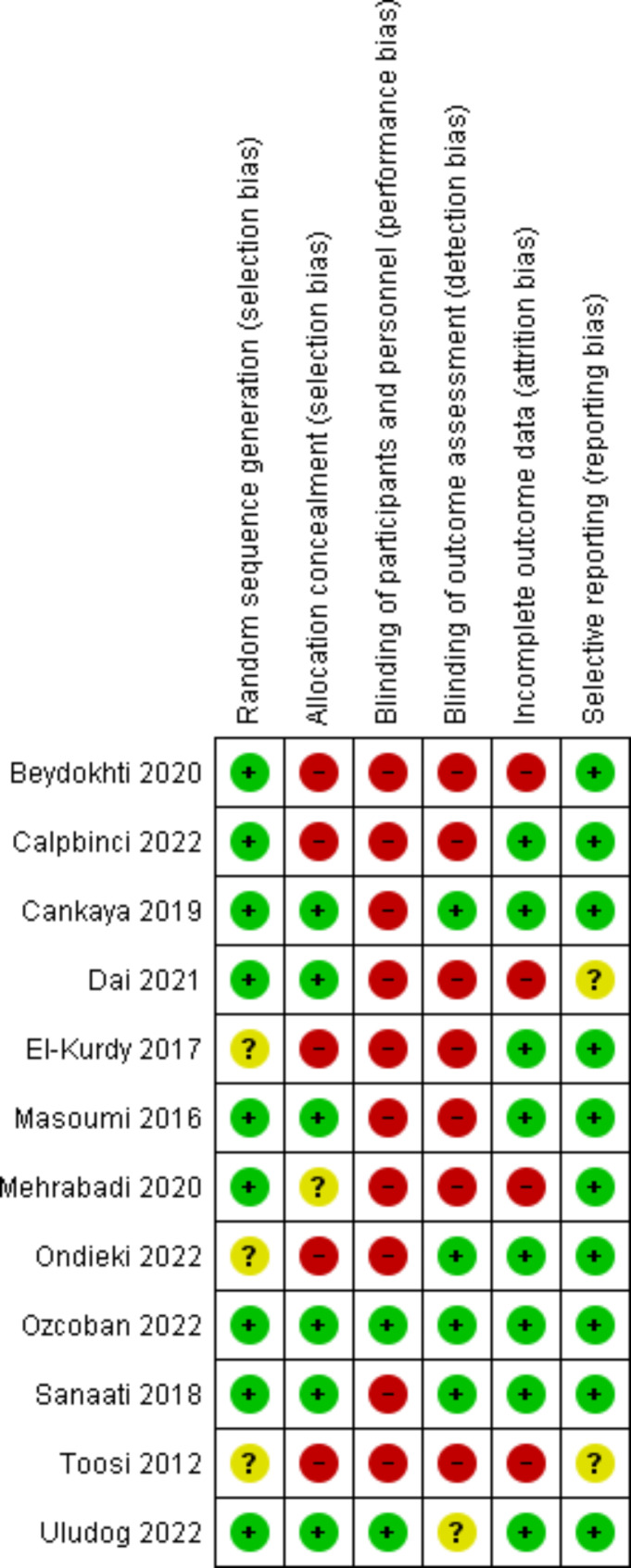
Risk of bias summary: Review authors’ judgments about each risk of bias item for each included study

The risk of bias was addressed in randomized controlled trials using the ROB-1 approach and in semi-experimental trials using the ROBINS-1 approach [30, 31]. Except for one case rated as unclear risk [22], all the randomized controlled trials were rated low risk in random sequence generation. Only six trials were rated as low risk in allocation concealment [28, 39, 40, 44, 47, 48], and the rest were rated as high or unclear risk. The nature of the studies made the blindness of the personnel providing the interventions impossible. Nevertheless, participants in only two studies were blinded regarding placement in the study groups and the type of intervention received in the other group [28, 47], and the rest were at high risk. In addition, only the outcome assessors in four studies were blinded [39, 46–48], and the rest were at high risk in this respect. Three studies were rated as high risk regarding incomplete outcome data or attrition bias [37, 40, 45], and the rest were low risk. Finally, all the studies were rated as low risk regarding selective reporting bias, and only two were rated as unclear risk [40, 50] (Table 3; Figs. 2 and 3).

**Table 3:** Risk of bias of included studies (RCTs)

Bias	Authors’ judgment	Support for judgment
**Beydokhti et al. (2020)**		
Random sequence generation	Low risk	Participants were allocated into interventions and control groups using the flip-the-coin method.
Allocation concealment	High risk	There was no evidence for allocation concealment.
Blinding of participants and personnel	High risk	Open-label design
Blinding of outcome assessors	High risk	Open-label design
Incomplete outcome data	High risk	Six of 66 participants in the intervention group (three due to not receiving allocated intervention and three due to Lost to follow-up) and one of 70 participants in the control group (due to Lost to follow-up) dropped out of the study.
Selective reporting	Low risk	A protocol is not available, but it is clear that all pre-specified and expected outcomes of interest are reported.
**Calpbinici & Özçirpan. (2022)**		
Random sequence generation	Low risk	The stratified block randomization method allocated participants into interventions and control groups.
Allocation concealment	High risk	There was no evidence for allocation concealment.
Blinding of participants and personnel	High risk	Open-label design
Blinding of outcome assessors	High risk	Open-label design
Incomplete outcome data	Low risk	23 of the 60 participants in the intervention group and 24 of the 60 participants in the control group dropped out of the study. Missing data were balanced across groups, and the reasons were similar.
Selective reporting	Low risk	A protocol is not available, but it is clear that all pre-specified and expected outcomes of interest are reported.
**Çankaya & Şimşek. (2019)**		
Random sequence generation	Low risk	Participants were allocated into interventions and control groups using the block randomization method and random numbers table.
Allocation concealment	Low risk	The randomization process was done by a statistical expert, not from among the authors.
Blinding of participants and personnel	High risk	No blinding.
Blinding of outcome assessors	Low risk	Outcome assessment and data analysis were done by someone who was not the study staff.
Incomplete outcome data	Low risk	Five of the 60 participants in the intervention group and Three of the 60 participants in the control group dropped out of the study, but the reasons for missing data were unrelated to the outcome.
Selective reporting	Low risk	A protocol is not available, but it is clear that all pre-specified and expected outcomes of interest are reported.
**Dai et al. (2021)**		
Random sequence generation	Low risk	A random number table was used for randomization assignment in this study
Allocation concealment	Low risk	An independent researcher did the randomization process.
Blinding of participants and personnel	High risk	Open-label design
Blinding of outcome assessors	High risk	Open-label design
Incomplete outcome data	High risk	12 of 38 participants in the intervention and 8 of 38 participants in the control group were excluded which were imbalanced in numbers or reasons
Selective reporting	Unclear risk	A protocol is not available,
**Kurdy et al. (2017)**		
Random sequence generation	Unclear risk	It is mentioned in the text that the groups are allocated randomly, but the authors needed to provide more information in this regard.
Allocation concealment	High risk	There was no evidence for allocation concealment.
Blinding of participants and personnel	High risk	Open-label design
Blinding of outcome assessors	High risk	Open-label design
Incomplete outcome data	Low risk	All data is present.
Selective reporting	Low risk	A protocol is not available, but it is clear that all pre-specified and expected outcomes of interest are reported.
**Masoumi et al. (2016)**		
Random sequence generation	Low risk	A random number table was used for randomization assignment in this study
Allocation concealment	Low risk	Allocation concealment was done by sequentially numbered, sealed, opaque envelopes
Blinding of participants and personnel	High risk	Open-label design
Blinding of outcome assessors	High risk	Open-label design
Incomplete outcome data	Low risk	5 of 80 participants in the intervention and 5 of 80 in the control group were excluded, balanced in numbers and reasons.
Selective reporting	Low risk	A protocol is available, and all pre-specified outcomes of interest to the review are reported in a pre-specified way.
**Mehrabadi et al. (2020)**		
Random sequence generation	Low risk	Participants were allocated into interventions and control groups using the block randomization method
Allocation concealment	Unclear risk	There needs to be more information in this regard.
Blinding of participants and personnel	High risk	Open-label design
Blinding of outcome assessors	High risk	Open-label design
Incomplete outcome data	High risk	10 of 66 participants in the intervention and 22 of 66 in the control group were excluded, which were imbalanced in numbers and reasons.
Selective reporting	Low risk	A protocol is available, and all pre-specified outcomes of interest to the review are reported in a pre-specified way.
**Ondieki et al. (2022)**		
Random sequence generation	Unclear risk	It is mentioned in the text that the groups are allocated randomly, but the authors needed to provide more information in this regard.
Allocation concealment	High risk	There was no evidence for allocation concealment.
Blinding of participants and personnel	High risk	Open-label design
Blinding of outcome assessors	Low risk	blinding
Incomplete outcome data	Low risk	Four of 60 participants in the intervention and six of 59 participants in the control group were excluded, but the reasons for missing data were unrelated to the outcome.
Selective reporting	Low risk	A protocol is available, and all pre-specified outcomes of interest to the review are reported in a pre-specified way.
**Ozcoban et al. (2022)**		
Random sequence generation	Low risk	Participants were allocated into interventions and control groups using a web-based computer application.
Allocation concealment	Low risk	Participants were randomly assigned to one of three groups by drawing lots
Blinding of participants and personnel	Low risk	Blinding was applied to participants in the three groups during allocation, intervention, and data. Collection. It was not possible to blind the researcher
Blinding of outcome assessors	Low risk	The second researcher, who did not know which group was which, led the data collection
Incomplete outcome data	Low risk	31 of 140 participants in the intervention and 7 of 80 in the control group were excluded, but the reasons for missing data were unrelated to the outcome.
Selective reporting	Low risk	A protocol is available, and all pre-specified outcomes of interest to the review are reported in a pre-specified way.
**Sanaati et al. (2018)**		
Random sequence generation	Low risk	Participants were allocated into interventions and control groups using the stratified block randomization method.
Allocation concealment	Low risk	Allocation concealment was done by sequentially numbered, sealed, opaque envelopes.
Blinding of participants and personnel	High risk	No blinding
Blinding of outcome assessors	Low risk	The outcomes assessor was blinded by using data coded for intervention groups.
Incomplete outcome data	Low risk	One of 63 participants in intervention group 1 and one of 63 participants in intervention group 2 were excluded, but the reasons for missing data were unrelated to the outcome.
Selective reporting	Low risk	A protocol is available, and all pre-specified outcomes of interest to the review are reported in a pre-specified way.
**Toosi et al. (2012)**		
Random sequence generation	Unclear risk	It is mentioned in the text that the groups are allocated randomly, but the authors needed to provide more information in this regard.
Allocation concealment	High risk	There was no evidence for allocation concealment.
Blinding of participants and personnel	High risk	Open-label design.
Blinding of outcome assessors	High risk	Open-label design
Incomplete outcome data	High risk	Two of the 44 participants in the intervention and two of 42 in the control group were excluded, but groups have not determined the reasons.
Selective reporting	Unclear risk	A protocol is not available.
**Uludag et al. (2022)**		
Random sequence generation	Low risk	Participants were allocated into intervention and control groups using the block randomization method, and blocks were selected using simple random sampling.
Allocation concealment	Low risk	Random selection of the blocks was performed by using numbers from a website producing randomization numbers.
Blinding of participants and personnel	Low risk	Experimental and control groups were blinded to their groups. However, it was not possible to blind the researchers.
Blinding of outcome assessors	Unclear risk	There needs to be more information in this regard.
Incomplete outcome data	Low risk	Six of 23 participants in the intervention and one of 21 participants in the control group were excluded, but the reasons for missing data were unrelated to the outcome, and the analysis was based on the intention-to-treat protocol.
Selective reporting	Low risk	A protocol is available, and all pre-specified outcomes of interest to the review are reported in a pre-specified way.

The overall risk of bias for the semi-experimental trials was considered serious because there was at least one serious bias in the investigated subdomains. Thus, all studies were at moderate to severe risk of bias due to confounders. One study was at a low risk of bias caused by the participants’ selection [49], and the rest were at a high risk. All the studies were low risk regarding biases caused by the classification of interventions and deviations from intended interventions. Only one study was at low risk regarding biases due to missing data [42], and the rest were at moderate to severe risk. All the studies were at high risk of bias due to the measurement of outcomes. Only one study was at low risk regarding biases caused by the selection of reported results [27], and the rest were at moderate to severe risk. In summary, all semi-experimental trials examined in this study were at high risk of bias (Table 4).

**Table 4:** Risk of bias of included studies (Semiexperimental study)

Author	Firouzbakht et al. (2015)	Gurkan & Ekşi (2017)	Isbir et al. (2017)	Karabulut et al. (2016)	Serçekuş & Başkale. (2015)	Taheri et al. (2014)
Bias due to confounding	Moderate	Moderate	Serious	Serious	Moderate	Moderate
Bias in the selection of participants	Serious	Serious	Serious	Serious	Serious	Low
Bias in the classification of interventions	Low	Low	Low	Low	Low	Low
Bias due to deviations from intended interventions	Low	Low	Low	Low	Low	Low
Bias due to missing data	Serious	Moderate	Low	Serious	Moderate	Moderate
Bias in the measurement of outcomes	Serious	Serious	Serious	Serious	Serious	Serious
Bias in the selection of reported result	Serious	Low	Moderate	Low	Moderate	Moderate
Overall	Serious	Serious	Serious	Serious	Serious	Serious

Low: Low risk of bias (the study is comparable to a well-performed randomized trial concerning this domain); Moderate: Moderate risk of bias (the study is sound for a non-randomized study concerning this domain but cannot be considered comparable to a well-performed randomized trial); Serious: Serious risk of bias (the study has some important problems)

## Outcome measurement

### Primary outcomes

#### Fear of childbirth

**Fig. 4:**
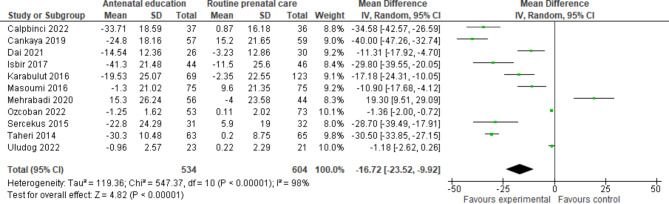
Antenatal education versus routine prenatal care, Outcome 1: Fear of childbirth

Eight randomized controlled [28, 38–40, 44–47] and four semi-experimental [12, 42, 43, 49] trials compared the fear of childbirth in pregnant women receiving prenatal education and routine care with women receiving only routine care. All studies have used the W-DEQ questionnaire to measure fear of childbirth, except four studies that had used a researcher-made questionnaire [44], The Fear of Birth Scale (FOBS) [28], Delivery Fear questionnaire [49], and the Fear of Childbirth questionnaire [47]. Calpbinici and Özçirpan [38] (MD -34.58, 95% CI -42.57 to -26.59, p < 0.001), Çankaya and Şimşek [39] (MD -40.00, 95% CI -47.26 to -32.74, p < 0.001), Dai et al. [40] (MD -11.31, 95% CI -17.92 to -4.70, p = 0.001), İsbir et al. [42] (MD -29.80, 95% CI -39.55 to -20.05, p < 0.01), Karabulut et al. [43] (MD -17.18, 95% CI -24.31 to -10.05, p = 0.022), Masoumi et al. [44] (MD -10.90, 95% CI -17.68 to -4.12, p = 0.007), Ondieki [46] (p < 0.001), Ozcoban et al. [47] (MD -1.36, 95% CI -2.00 to -0.72, p = 0.036), Serçekuş and Başkale [12] (MD -28.70, 95% CI -39.49 to -17.91, p < 0.001), and Taheri et al. [49] (MD -30.50, 95% CI -33.85 to -27.15, p < 0.001) indicated that prenatal education significantly decrease the fear of childbirth. However, Mehrabadi et al. concluded that prenatal education significantly increases the fear of childbirth (MD 19.30, 95% CI 9.51 to 29.09, p < 0.001) [45]. Researchers had access to data on changes in the average scores of fear of childbirth before and after the intervention in control and intervention groups in all studies except for Ondieki’s study. All the data were entered into the meta-analysis. The data from 11 studies conducted on 1138 pregnant women showed that prenatal education and routine care might essentially decrease fear of childbirth among pregnant mothers compared to those receiving only routine care (SMD − 16.7, 95% CI -23.5 to -9.9, p < 0.00001, 11 trials, 1138 women, Low certainty) (Fig. 4).

#### Pain intensity in the first labour phase

**Fig. 5:**
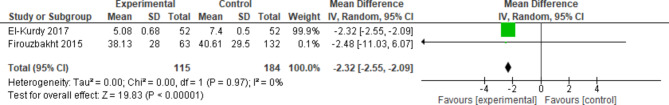
Antenatal education versus routine prenatal care, Outcome 2: Pain intensity in the first labour phase

A randomized controlled trial [22] and a semi-experimental trial [41] have compared the pain intensity in the first labour phase in women receiving prenatal education and routine care with women receiving only routine care. El-Kurdy et al. [22] used Numerical Rating Scale to measure pain intensity in the first labour stage, and Firouzbakht et al. [41] used Visual Analog Scale. El-Kurdy et al. and Firouzbakht et al. showed that prenatal education and routine care decrease the pain intensity in the first labour phase in pregnant women compared to women receiving only routine care. However, although the results of the former were significant (MD -2.32, 95% CI -2.55 to -2.09, p < 0.001), the latter was insignificant (MD -2.48, 95% CI -11.03 to 6.07, p = 0.58). The meta-analysis applied to the data from two studies conducted on 299 pregnant women showed that prenatal education and routine care compared to routine care alone decrease the pain intensity in the first labour phase (SMD − 2.3, 95% CI -2.5 to -2.1; p < 0.00001, 2 trials, 299 women, Moderate certainty) (Fig. 5).

#### Pain intensity in the second labour phase

**Fig. 6:**
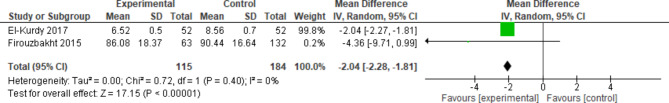
Antenatal education versus routine prenatal care, Outcome 3: Pain intensity in second

A randomized controlled trial [22] and a semi-experimental trial [41] have compared the pain intensity in the second labour phase in women receiving prenatal education and routine care with women receiving only routine care. El-Kurdy et al. [23] used Numerical Rating Scale to measure pain intensity in the second labour phase[22], and Firouzbakht et al. [41] used Visual Analog Scale. El-Kurdy et al. and Firouzbakht et al. showed that providing prenatal education along with routine care decreased the pain intensity in the second labour phase in pregnant women, with the difference that the former decrease was significant (MD -2.04, 95% CI -2.27 to -1.81, p < 0.001). Still, the latter was insignificant (MD -4.36, 95% CI -9.71 to 0.99, p = 0.58). The meta-analysis applied to data from two studies conducted on 299 pregnant women showed that providing prenatal education and routine care compared to providing only routine care decreased pain intensity in the second labour phase (SMD − 2.0, 95% CI -2.3 to -1.8, p < 0.00001, 2 trials, 299 women, Moderate certainty) (Fig. 6).

#### Childbirth experience

We found no randomized controlled or semi-experimental trials comparing the birth experience in women receiving prenatal education and routine care with women receiving only routine care.

### Secondary outcomes

#### Maternal attachment

A randomized controlled trial [50] and a semi-experimental trial [12] have compared maternal attachment in women receiving prenatal education and routine care with those receiving only routine care. Toosi et al. [50] used Cranley’s Mother-Fetal Attachment Scale and showed that providing prenatal education and routine care significantly increases maternal-fetal attachment during pregnancy (MD 2.9, 95% CI 0.45 to 5.35, p = 0.023). Serçekuş and Başkale [12] used the Maternal Attachment Inventory four months after delivery and the Postnatal Paternal-Infant Attachment Questionnaire six months after delivery. They showed that the effect of prenatal education and routine care on maternal and parental-fetal attachments is not significant in the postpartum period (respectively MD 1.6, 95% CI -1.1 to 4.5, p = 0.258 and MD 0.5, 95% CI -2.61 to 3.61, p = 0.625). Since these two studies have evaluated different outcomes, no meta-analysis was performed on them.

#### Postpartum depression

**Fig. 7:**
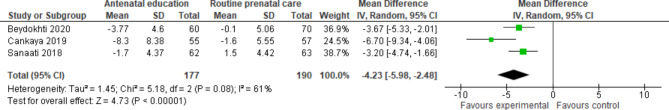
Antenatal education versus routine prenatal care, Outcome 4: Postpartum depression

Three randomized controlled trials [37, 39, 48] and one semi-experimental trial [27] have compared postpartum depression in women receiving prenatal education and routine care with those receiving only routine care. To evaluate postpartum depression, Beydokhti et al. [37] and Sanaati et al. [48] used the Edinburgh Postnatal Depression Scale (EPDS), and Çankaya and Şimşek used the Depression Anxiety and Stress Scale (DASS-21). The three mentioned studies measured the depression score of pregnant women before providing prenatal education in the intervention and control groups and also six weeks after delivery. Beydokhti et al. [37] (MD -3.67, 95% CI -5.33 to -2.01, p = 0.001), Sanaati et al. [48] (MD -3.20, 95% CI -4.74 to -1.66, p < 0.001), and Çankaya and Şimşek [39] (MD -6.70, 95% CI -9.34 to -4.06, p < 0.001) showed a significant decrease in the mean score of depression after providing prenatal education to the intervention groups compared to the control group. The meta-analysis applied to data from three studies conducted on 367 pregnant women showed that providing prenatal education and routine care compared to providing only routine care may decrease postpartum depression (SMD − 4.23, 95% CI -5.98 to -2.48; 3 trials, 367 women, Low certainty) (Fig. 7).

Gürkan et al. [27] performed a semi-experimental study without examining the depression scores of pregnant women before receiving prenatal education and measured their depression scores using the EPDS approach. They compared these scores with women receiving prenatal education and routine care six weeks after delivery and found no significant difference (MD -0.4, 95% CI -3.32 to 2.52, p > 0.05). Due to its different study design, this study was excluded from the meta-analysis.

#### Postpartum anxiety

Two randomized controlled trials [39, 48] compared postpartum anxiety in women receiving prenatal education and routine care with those receiving only routine care. Çankaya and Şimşek [39] used the DASS-21 approach to measure postpartum anxiety. They found a significant difference between the average anxiety score before providing prenatal education and six weeks after delivery (MD -04.6, 95% CI -7.12 to -2,08, p < 0.001). Sanaati et al. [48] evaluated postpartum anxiety using Spielberger State-Trait Anxiety Inventory based on two approaches (i.e., State anxiety and Trait anxiety) in women receiving prenatal education and routine care and those receiving only routine care. They found a significant decrease between the average state anxiety score before providing prenatal education and six weeks after delivery (MD -4.66, 95% CI -6.74 to -2.59, p = 0.001). In addition, they found a significant decrease in the average score of trait anxiety before providing prenatal education and six weeks after delivery (MD -5, 95% CI -8.4 to -1.6, p = 0.013). These two studies were excluded from the meta-analysis because they had different intended outcomes.

**Fig. 8:**
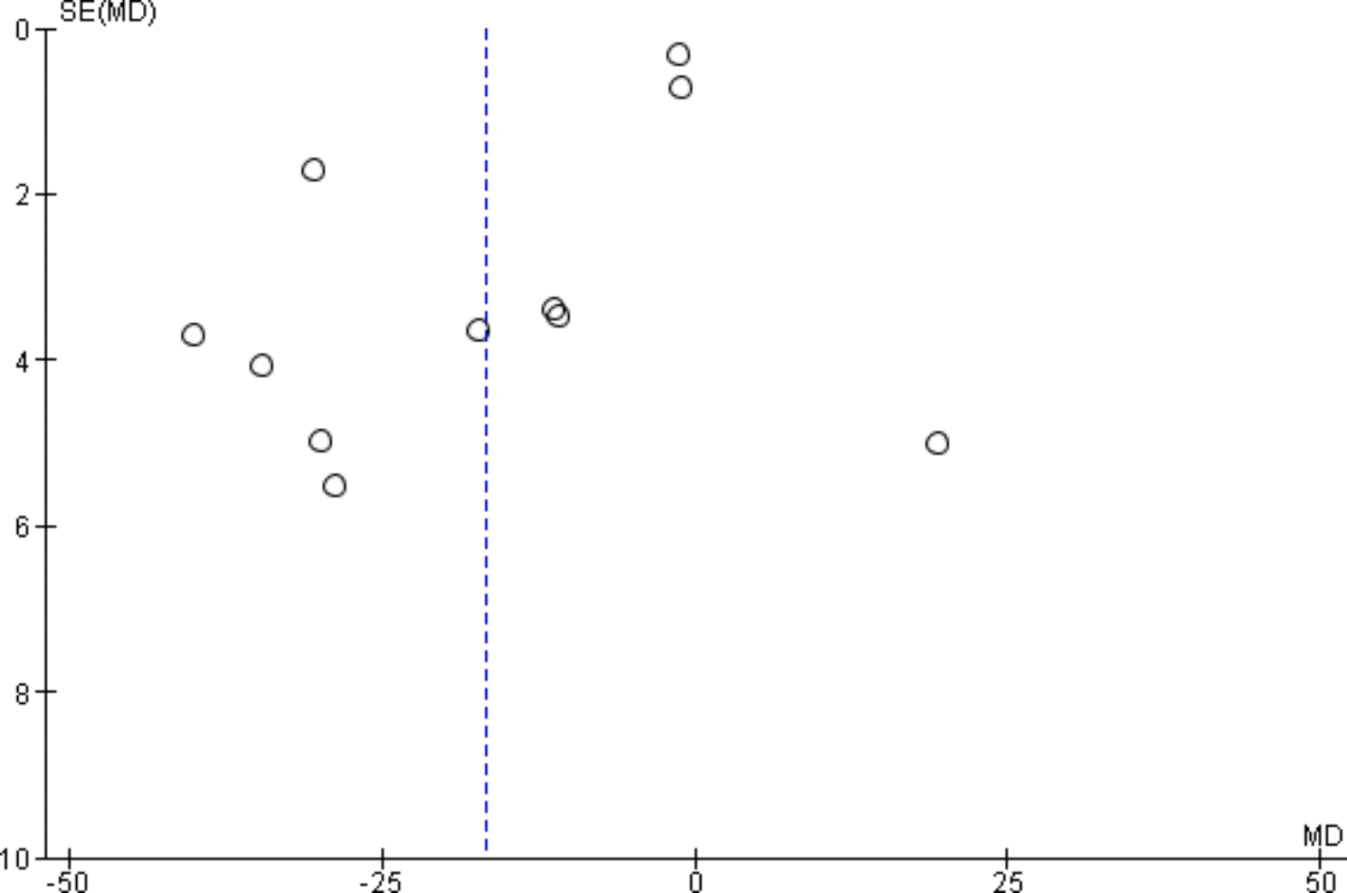
Funnel plot, Outcome 1: Fear of childbirth

There were 11 studies on the fear of childbirth. Thus, publication bias only for this outcome was investigated using a funnel plot. Since the funnel plot was relatively symmetrical for this outcome, its publication bias was considered not serious (Fig. 8).

Examining the quality or certainty of the evidence using the GRADE approach showed that the quality of evidence for fear of childbirth and postpartum depression due to serious concerns in evaluating the risk of bias and inconsistency was reduced by two degrees and reached the level of low certainty. However, the quality of evidence for the pain intensity in the first and second labour phases due to serious concern only in assessing the risk of bias was reduced by one degree and reached the level of moderate certainty (Table 5).

**Table 5:** Certainty of the evidence using the GRADE approach by outcomes

No of studies	Design	Risk of bias	Inconsistency	Indirectness	Imprecision	Publication bias	Antenatal education	Routine prenatal care	Pooled effect Relative (95% CI)	Final judgment
Fear of childbirth										
11	RCT Semi-experimental	Serious	Serious	No serious	No serious	No serious	534	604	SMD 16.7 lower (23.5 lower to 9.9 lower)	⊕⊕⊖⊖ low
Pain intensity in the first labour phase										
2	RCT^*^ Semi-experimental	Serious	No serious	No serious	No serious	No serious	115	184	SMD 2.3 lower (2.5 lower to 2.1lower)	⊕⊕⊕⊖ Moderate
Pain intensity in the second labour phase										
2	RCT^*^ Semi-experimental	Serious	No serious	No serious	No serious	No serious	115	184	SMD 2.0 lower (2.3 lower to 1.8 lower)	⊕⊕⊕⊖ Moderate
Postpartum depression										
4	RCT^*^ Semi-experimental	Serious	Serious	No serious	No serious	No serious	208	224	SMD 4.2 lower (5.9 lower to 2.5 lower)	⊕⊕⊖⊖ Low
**GRADE**: Grading of Recommendations Assessment, Development and Evaluation; **CI**: confidence interval; **RCT**: randomized controlled trial; **SMD**: standardized mean difference										

**GRADE Working Group grades of evidence**

**High certainty**: We are very confident that the true effect is close to the effect estimate.

**Moderate certainty**: We are moderately confident in the effect estimate; the true effect is likely to be close to the estimate of the effect, but there is a possibility that it is substantially different.

**Low certainty**: Our confidence in the effect estimate is limited; the true effect may be substantially different from the estimate of the effect.

**Very low certainty**: We have very little confidence in the effect estimate; the true effect is likely to be substantially different from the estimate of the effect.

## Discussion

This scoping review using a systematic approach included 12 randomized controlled trials and six semi-experimental trials investigating the effect of prenatal education on some maternal outcomes. We found no study investigating the outcomes of the childbirth experience. However, two studies investigating postpartum anxiety and maternal attachment were excluded from the meta-analysis due to their different approaches to examining the intended outcomes. We found two studies that have compared the labour pain intensity in women receiving prenatal education and routine care with women receiving only routine care. Considering that the studies evaluating pain intensity were consistent in terms of participants, interventions, and outcomes and also in the direction of effect size, they entered the meta-analysis [59, 60]. There was relatively good consistency in studies regarding outcomes of fear of childbirth and postpartum depression. Thus, a meta-analysis was performed on these outcomes. However, the low quality of the evidence on the desired outcomes in these studies was rated low to moderate, which prevents making definite conclusions regarding the effect of prenatal education on these outcomes. Nevertheless, it can be said that prenatal education may essentially decrease the fear of childbirth, postpartum depression, and pain intensity in the first and second stages of labour in pregnant mothers.

This review examined the risk of bias based on 18 trials and found that the overall risk of bias was high. Most randomized controlled trials included less than 200 participants in control and intervention groups, and their risk of bias was severe due to not blinding the participants and outcome assessors. Therefore, more extensive trials with fewer methodological limitations must report their results according to CONSORT principles [61] to provide more definitive conclusions in future systematic reviews.

Gagnon and Sandall [15] systematically reviewed the effect of individual and group training during pregnancy on information acquisition, anxiety, sense of control, labour pain, labour and delivery supports, breastfeeding, ability to newborn care, and psychological and social adaptation. They examined nine trials, including 2284 pregnant women. Studies reviewed by this paper needed more details regarding the randomization process, allocation concealment, and missing data, and the sample size was small to medium; thus, their internal validity was low. These studies lacked information regarding anxiety, social support, and breastfeeding and have examined outcomes such as information acquisition, the feeling of control, newborn care abilities, and some labour and delivery outcomes. Thus, the low quality of the included studies prevented us from drawing definitive conclusions. Brixval et al. [62] investigated the effect of prenatal education in small groups on midwifery and psychosocial outcomes of childbirth. It included seventeen trials that were different regarding the conditions of the intervention and control groups, reporting outcomes in a few trials, the heterogeneity of the studies in terms of interventions and outcomes, and the high risk of bias. Thus, it failed to draw definitive conclusions about the effect of prenatal education before the delivery.

Demirci et al. [63] reviewed the effect of prenatal education on the self-efficacy of childbirth in pregnant women. It included seven eligible articles, but the quality of evidence showed high heterogeneity between studies. The results of the meta-analysis showed that prenatal education had a positive and significant effect on outcome expectancy and efficacy expectancy.

Leutenegger et al. [64] investigated the effect of breathing and relaxation techniques taught in prenatal classes on maternal and neonatal outcomes such as mothers’ satisfaction with the labour and delivery, pain level, need for pharmacologic support for pain management, mobility in labour, delivery type, need to take blood from the baby to measure blood pH and 5-minute Apgar score. This study included nine randomized controlled trials and one semi-experimental study. The training provided in prenatal classes of these papers was very diverse and inconsistent, weakening the quality of this study’s evidence. The results showed that breathing and relaxation techniques might positively affect self-efficacy, and the need for medical support, especially epidural anaesthesia and labour pain, but no effect was seen on neonatal outcomes. Women who participated in the prenatal education class with breathing and relaxation techniques seemed to benefit from this intervention.

Hong et al. [65] investigated the effect of various prenatal education programs on maternal and neonatal physical and psychological outcomes to help the development of future guidelines for maternal and neonatal health. It included 14 controlled trials and nine observational studies. Except for the rate of cesarean delivery and the use of epidural anaesthesia, which were lower in prenatal education, the mothers’ physical outcomes were not significantly different. Stress and self-efficacy in the group receiving prenatal education (as outcomes of the mother’s mental health) were significantly improved, but no significant difference was observed in anxiety and depression. Neonatal outcomes such as weight at birth or gestational age at birth were the same between groups.

Pregnancy and puerperium are known as periods along with physical, personal, emotional, and social changes for women. Mood disorders related to these periods have been widely described, so women may represent a vulnerable population who might be severely psychologically affected. Recently published studies confirmed a significantly increased rate of depressive symptoms, anxiety, and thoughts of self-harm in the obstetric population after the declaration of the COVID-19 pandemic, even in subgroups generally at low risk [66]. So the importance of providing interventions to prevent psychological disorders is even more obvious.

### Strengths and limitations

Considering that the trials were inconsistent regarding interventions, contents, the studied populations, and the results evaluation method. Thus, comparing them was difficult and did not lead to definitive conclusions.

We found very few or no studies that have compared the labour pain intensity, childbirth experience, postpartum anxiety, and maternal attachment in women receiving prenatal education and routine care with women receiving only routine care.

### Implications for research

This scoping review using a systematic approach showed that there are very few or no studies on the effect of prenatal education on outcomes such as labour pain intensity, childbirth experience, postpartum anxiety, and maternal attachment. Therefore, high-quality trials with a more extensive sample size are suggested to clarify the effect of prenatal education on these outcomes. Our investigations showed that these studies lack sufficient quality to make definitive conclusions regarding the effect of prenatal education on other outcomes. Therefore, high-quality, randomized trials with a more extensive sample size are suggested to provide clear reports to make definitive decisions. In addition, future trials should consider the feasibility of prenatal education to make educational program development and evaluation possible.

## Conclusion

Our scoping review using a systematic approach and meta-analysis showed that providing prenatal education and routine care compared to providing only routine care may essentially decrease the fear of childbirth, postpartum depression, and pain intensity in the first and second stages of childbirth in pregnant mothers. However, our results do not provide definitive conclusions regarding the outcome of the childbirth experience, maternal-fetal attachment, and postpartum anxiety.

## Electronic supplementary material

Below is the link to the electronic supplementary material.

Supplementary Material 1

## Data Availability

All data are included in the tables.

## Abbreviations

WHO: World Health Organization
ACOG: American College of Obstetricians and Gynecologists
PROSPERO: International Prospective Register of Systematic Reviews
ROB-1: the risk of bias-1 approach
GRADE: Grading of Recommendations Assessment, Development and Evaluation
SMDs: standardized mean differences
FOBS: Fear of Birth Scale
EPDS: Edinburgh Postnatal Depression Scale
DASS-21: Depression Anxiety and Stress Scale
