# Supplementary material for: The effect of prenatal education on fear of childbirth, pain intensity during labour and childbirth experience: a scoping review using systematic approach and meta-analysis
Source: BMC Pregnancy Childbirth. 2023 Jul 27;23:541. doi: 10.1186/s12884-023-05867-0 (PMC10373291; doi:10.1186/s12884-023-05867-0)
Supplement: Supplementary file 1 — Supplementary Material 1 [file 12884_2023_5867_MOESM1_ESM.docx]

**Search strategy**

**PubMed**

1. ((((((((antenatal[Text Word]) OR (prenatal[Text Word])) OR (pregnancy[Text Word])) OR (birth[Text Word])) OR (childbirth[Text Word])) OR (labor[Text Word])) OR (obstetric[Text Word])) OR (delivery[Text Word])) OR (deliveries[Text Word])

2. ((((((education[Text Word]) OR ("parent education*"[Text Word])) OR (preparation[Text Word])) OR ("parent preparation"[Text Word])) OR ("early intervention"[Text Word])) OR (training[Text Word])) OR ("parent training"[Text Word]) AND ((randomizedcontrolledtrial[Filter]) AND (humans[Filter]))

3. 1 AND 2

4. ((((fear[Text Word]) OR (tokophobia[Text Word])) OR ("fear of childbirth"[Text Word])) OR ("stress disorder*"[Text Word])) OR ("fear of birth"[Text Word]) AND ((randomizedcontrolledtrial[Filter]) AND (humans[Filter]))

5. (((birth[Text Word]) OR (childbirth[Text Word])) OR (delivery[Text Word])) OR (labor[Text Word]) AND ((randomizedcontrolledtrial[Filter]) AND (humans[Filter]))

6. 4 AND 5

7. 3 AND 6

8. (((((pain[Text Word]) OR (ache[Text Word])) OR (suffering[Text Word])) OR ("labor pain"[Text Word])) OR ("obstetric pain"[Text Word])) OR ("delivery pain"[Text Word]) AND ((randomizedcontrolledtrial[Filter]) AND (humans[Filter]))

9. ((((labor[Text Word]) OR (contraction*[Text Word])) OR (birth[Text Word])) OR (childbirth[Text Word])) OR (delivery[Text Word])

10. 8 AND 9

11. 3 AND 10

12. (((((("mood disorder*"[Text Word]) OR ("depressive disorder*"[Text Word])) OR (depression[Text Word])) OR ("depressive symptom*"[Text Word])) OR (anxiety[Text Word])) OR ("anxiety disorder*"[Text Word])) OR ("psychological disorder*"[Text Word]) AND ((randomizedcontrolledtrial[Filter]) AND (humans[Filter]))

13. ((((((postpartum[Text Word]) OR (puerperium[Text Word])) OR (pregnancy[Text Word])) OR (gestation[Text Word])) OR (antenatal[Text Word])) OR (prenatal[Text Word])) OR (postnatal[Text Word])

14. 12 AND 13

15. 3 AND 14

16. (((((((((((((("childbirth experience*"[Text Word]) OR ("experience of childbirth*"[Text Word])) OR ("birth experience*"[Text Word])) OR ("experience of birth*"[Text Word])) OR ("labor experience*"[Text Word])) OR ("experience of labor*"[Text Word])) OR ("delivery experience*"[Text Word])) OR ("experience of delivery*"[Text Word])) OR ("maternal experience*"[Text Word])) OR ("experience of mother*"[Text Word])) OR ("mother’s experience"[Text Word])) OR ("birth perception*"[Text Word])) OR ("perception of birth*"[Text Word])) OR ("childbirth perception*"[Text Word])) OR ("perception of childbirth*"[Text Word]) AND ((randomizedcontrolledtrial[Filter]) AND (humans[Filter]))

17. 3 AND 16

18. (((((attachment[Text Word]) OR (bonding[Text Word])) OR ("maternal fetal attachment"[Text Word])) OR ("maternal fetal bonding"[Text Word])) OR ("mother infant attachment"[Text Word])) OR ("mother infant bonding"[Text Word])

19. 3 AND 18

**Web of Science**

1. ((((((((TS=(antenatal)) OR TS=(prenatal)) OR TS=(pregnancy)) OR TS=(birth)) OR TS=(childbirth)) OR TS=(labo*r)) OR TS=(obstetric)) OR TS=(delivery)) OR TS=(deliveries)

2. ((((((TS=(education)) OR TS=("parent education*" )) OR TS=(preparation)) OR TS=("parent preparation" )) OR TS=("early intervention")) OR TS=(training)) OR TS=("parent training")

3. ((TS=("randomi* control* trial*" )) OR TS=("randomi* trial*" )) OR TS=("randomi* clinical trial*")

4. #1 AND #2 AND #3

5. (((TS=(fear)) OR TS=(tokophobia)) OR TS=("stress disorders")) OR TS=("fear of childbirth")

6. ((((TS=(birth)) OR TS=(childbirth)) OR TS=(parturition)) OR TS=(delivery)) OR TS=(labo*r)

7. #5 AND #6

8. #4 AND #7

9. (((((TS=(pain)) OR TS=(ache)) OR TS=(suffering)) OR TS=("labo*r pain")) OR TS=("obstetric pain")) OR TS=("delivery pain")

10. ((((TS=(labo*r)) OR TS=(birth)) OR TS=(childbirth)) OR TS=(contraction*)) OR TS=(delivery)

11. #10 AND #9

12. #4 AND #11

13. ((((((TS=("mod disorder*")) OR TS=("depressive disorder*")) OR TS=(depression)) OR TS=("depressive symptom*")) OR TS=(anxiety)) OR TS=("anxiety disorder*")) OR TS=("psychological diorder*")

14. (((((((TS=(postpartum)) OR TS=(puerperium)) OR TS=(pregnancy)) OR TS=(gestation)) OR TS=(post*birth)) OR TS=(antenatal)) OR TS=(prenatal)) OR TS=(postnatal)

15. #13 AND #14

16. #4 AND #15

17. ((((((((((((((TS=(“childbirth experience*” )) OR TS=(“experience of childbirth*” )) OR TS=(“birth experience*” )) OR TS=("experience of birth*” )) OR TS=(“labour experience*” )) OR TS=(“experience of labour*” )) OR TS=(“delivery experience*” )) OR TS=(“experience of delivery*” )) OR TS=(“maternal experience*” )) OR TS=(“experience of mother*” )) OR TS=(“mother’s experience” )) OR TS=(“birth perception*” )) OR TS=(“perception of birth*” )) OR TS=(“childbirth perception*” )) OR TS=(“perception of childbirth*”)

18. #17 AND #4

19. (((((TS=(attachment)) OR TS=(bonding)) OR TS=("maternal f*etal bonding")) OR TS=("maternal f*etal attachment")) OR TS=("mother infant bonding")) OR TS=("mother infant attachment")

20. #19 AND #4

**Cochrane**

1. (antenatal or prenatal or labor or pregnancy or delivery or obstetric or childbirth or birth).ti,kw,ab.

2. (education or "parent education*" or preparation or "early intervention" or training).ti,kw,ab.

3. ("randomi* control* trial*" or "randomi* trial*" or "randomi* clinical trial*").ti,kw,ab.

4. 1 and 2 and 3

5. (fear or tokophobia or "fear of childbirth" or "fear of birth" or "stress disorder*").ti,kw,ab.

6. (birth or childbirth or delivery or labor).ti,kw,ab.

7. 5 and 6

8. 4 and 7

9. (pain or "labor pain" or suffering or ache or "obstetric pain or delivery pain").ti,kw,ab.

10. (labor or labour or contraction or delivery or birth or childbirth).ti,kw,ab.

11. 9 and 10

12. 4 and 11

13. ("mood disorder" or "depressive disorder*" or depression or "depressive symptom" or anxiety or " anxiety symptom").ti,kw,ab.

14. (postpartum or puerperium or pregnancy or gestation or prenatal or antenatal or postnatal).ti,kw,ab.

15. 13 and 14

16. 4 and 15

17. ("chlidbirth experience" or " experience of childbirth" or "birth experience" or " experience of birth" or "maternal experience" or "experience of mother*" or "mother`s experience" or "labor experience" or "experience of labor" or "delivery experience" or "experience of delivery" or "birth perception" or "perception of birth" or "childbirth perception" or "perception of childbirth").ti,kw,ab.

18. 4 and 17

19. (attachment or bonding or "maternal f*etal attachment" or "mother infant attachment" or "maternal f*etal bonding" or "mother infant bonding").ti,kw,ab.

21. 4 and 19
